# Pathological, Morphometric and Correlation Analysis of the Modified Mankin Score, Tidemark Roughness and Calcified Cartilage Thickness in Rat Knee Osteoarthritis after Extracorporeal Shockwave Therapy

**DOI:** 10.7150/ijms.67741

**Published:** 2022-01-01

**Authors:** Jai-Hong Cheng, Wen-Yi Chou, Ching-Jen Wang, Ka-Kit Siu, Jei-Ming Peng, Yi-No Wu, Meng-Shiou Lee, Chien-Yiu Huang, Jih-Yang Ko, Shun-Wun Jhan

**Affiliations:** 1 Center for Shockwave Medicine and Tissue Engineering, Kaohsiung Chang Gung Memorial Hospital and Chang Gung University College of Medicine, Kaohsiung, Taiwan.; 2 Department of Medical Research, Kaohsiung Chang Gung Memorial Hospital and Chang Gung University College of Medicine, Kaohsiung, Taiwan.; 3 Department of Leisure and Sports Management, Cheng Shiu University, Kaohsiung, Taiwan.; 4 Department of Orthopedic Surgery, Kaohsiung Chang Gung Memorial Hospital and Chang Gung University College of Medicine, Kaohsiung, Taiwan.; 5 Park One International Hospital, Kaohsiung, Taiwan.; 6 Institute for Translational Research in Biomedicine, Kaohsiung Chang Gung Memorial Hospital and Chang Gung University College of Medicine, Kaohsiung, Taiwan.; 7 School of Medicine, Fu Jen Catholic University, New Taipei City, Taiwan.; 8 Department of Chinese Pharmaceutical Science and Chinese Medicine Resources, China Medical University, 91, Hsueh-Shih Road, Taichung, Taiwan.

**Keywords:** osteoarthritis, extracorporeal shockwave therapy, tidemark roughness, calcified cartilage thickness, statistical analysis.

## Abstract

The paper displayed the pathological changes and relationships of the modified Mankin score, tidemark roughness and calcified cartilage (CC) thickness by extracorporeal shockwave therapy (ESWT) (0.25 mJ/ mm^2^ with 800 impulses) on different positions of the medial and lateral rat knee OA joint. After the experiments, the articular cartilage was assessed using histomorphometry, image analysis and statistical method. In the micro-CT analysis, ESWT on medial groups were better than lateral groups in the trabecular volume and trabecular number. The data showed a strong negative correlation between the modified Mankin score and tidemark roughness (r = -0.941; P < 0.001). In terms of the relationship of tidemark roughness with CC thickness, the medial and Sham groups showed a significant negative correlation (r = -0.788, P = 0.022). Additionally, the Euclidean distance derived from 3D scatter plot analysis was an indicator of chondropathic conditions, exhibiting a strong correlation with OA stage in the articular cartilage of the femur (r = 0.911, P < 0.001) and tibia (r = 0.890, P < 0.001) after ESWT. Principle component analysis (PCA) further demonstrated that ESWT applied to medial locations had a better outcome than treatment at lateral locations for knee OA by comparing with Sham and OA groups, and CC thickness was the most important factor affecting hyaline cartilage repair after ESWT.

## Introduction

Osteoarthritis (OA) is a most comment type of joint disease that caused damage to infrapatellar fat pad, synovial membrane, meniscus, articular cartilage and subchondral bone 1-3. According to the etiology of disease, OA has been classified as primary or idiopathic and secondary OA 4, 5. Primary OA (degenerative OA) is a result of the aging to occur degenerative changes in the joint 4. Secondary OA (post-traumatic OA; PTOA) is normally associated with trauma and leading to articular cartilage fractures, meniscal tears, chondral lesions and ligamentous injuries in the joint 6. OA often occurs in the hip, knee and ankle joints. Several factors have been reported to affect the incidence of this disease, including gender, overuse of joints, bone marrow density, obesity, trauma and degeneration 7. These factors induce damage owing to wear and tear of the articular surface and further damage to the subchondral bone in the joint. All damaged tissues induce inflammation in the joint, causing pain symptoms 8. Further, intra-articular fractures, subchondral sclerosis and osteophyte formation may be observed in the affected joint. In OA animal model, the surgical method is used to destabilize of joint by anterior cruciate ligament transection and meniscectomy (ACLT+MMx) to create knee OA 9. This model is convenience to observe the progression of OA disease. The pathological changes in OA animal model are displayed including, cartilage degradation, subchondral osteopenia followed by sclerosis, and osteophyte formation 9-11.

The cellular organization differs in different zones of the articular cartilage, including the tangential zone (superficial), transitional zone (intermediate), radial zone (deeper) and calcified cartilage (CC) 12-14. CC consists of the deeper layers of articular cartilage in the joint, and is involved in nutrient transportation and transfer of mechanical forces between the subchondral bone and the articular cartilage 14. The tidemark is the interface between calcified and uncalcified cartilage in the joint. Tidemark advancement and duplication are affected by cartilage thinning and subchondral bone thickening during OA progression 15, 16. Morphological changes of CC, including CC thickening, tidemark duplication and tidemark roughness, are all associated with OA 17, 18. Currently, a literature review is summarized the OA drug targets which are required for further investigation in the preclinical and clinical experiments such as matrix metalloproteinase-inhibitor and bone morphogenetic protein 7 for cartilage; zoledronic acid and Risedronate for subchondral bone; IL-1 receptor antagonist and lutikizumab for inflammatory processes; tanezumab and mavatrep for pain processes; and cox-2 inhibitor and metformin for pain and for reducing the risk of joint replacement 19.

Extracorporeal shockwave therapy (ESWT) is a noninvasive mechanotherapy by which to promote tissue regeneration through anti-inflammatory effects, angiogenesis, osteogenesis and cell proliferation in soft and hard tissues 20-22. Many molecular pathways are reported to induce by ESWT such as focal adhesion kinase, bone morphogenetic proteins (BMPs), Wingless-related integration sites (Wnts), vascular endothelial growth factor (VEGF), insulin-like growth factor 1 (IGF1) and Toll-like receptor 3 (TLR3) signaling pathway 21, 22. This therapy has been reported to have chondroprotective effects in a rat knee OA model and human clinical trials 23-26. Nevertheless, no specific locations of ESWT have been systemically identified as being most beneficial, and there has been no discussion or comparison of the positions which ESWT is application for knee OA. In a previous study, we compared different sites of ESWT application for the treatment of knee OA, and found that the best sites were the medial femur and the tibia condyles 27, 28. However, detailed analysis of the pathological changes of tidemark roughness and CC thickening of the knee after ESWT has not been performed and discussed. We therefore aimed to elucidate in detail the changes in tidemark roughness and CC thickness, as well their correlations with the modified Mankin score, after ESWT at different sites of the knee for the treatment of OA.

## Materials and Methods

### Animals

One hundred rats (Sprague-Dawley, six weeks old, BioLASCO, Taipei, Taiwan) were used in the experiments. The rats were maintained in the Center for Laboratory Animals for 1 week before the induction of OA. All rats were housed at 23 ± 1°C, humidity at 50 % ± 20 %, under light on at 5 am and light off at 5 pm for a 12-hour light and dark cycle and given food and water. Treatment of all rats in the study followed the Institutional Animal Care and Use Committee (IACUC) protocol, and the experiments were approved by the Animal Care Committee of Kaohsiung Chang Gung Memorial Hospital and the permission number is 2014082801.

### Induction of the rat knee OA model

For OA induction, the rats were anesthetized with the injection of Rompun (5mg/kg/dose)-Zoletil (20mg/kg/dose) mixture intramuscularly. The left knee was prepared and draped in surgically sterile fashion. The left knee was prepared and a straight anterior skin incision was made. Then the left knee joint was opened through medial parapatellar arthrotomy. Underwent a mini-arthrotomy and transection of the anterior cruciate ligament (ACL) of the left knee, and medial meniscectomy (MMx) was performed by excising the entire medial meniscus, as described in previous studies 27, 28. Prophylactic antibiotic treatment with ampicillin (50 mg/kg body weight) was administered for 5 days after surgery. Postoperatively, the animals were cared by a veterinarian, and the surgical sites were examined and the activity of the animals observed daily.

### Shockwave application

**Figure 1:**
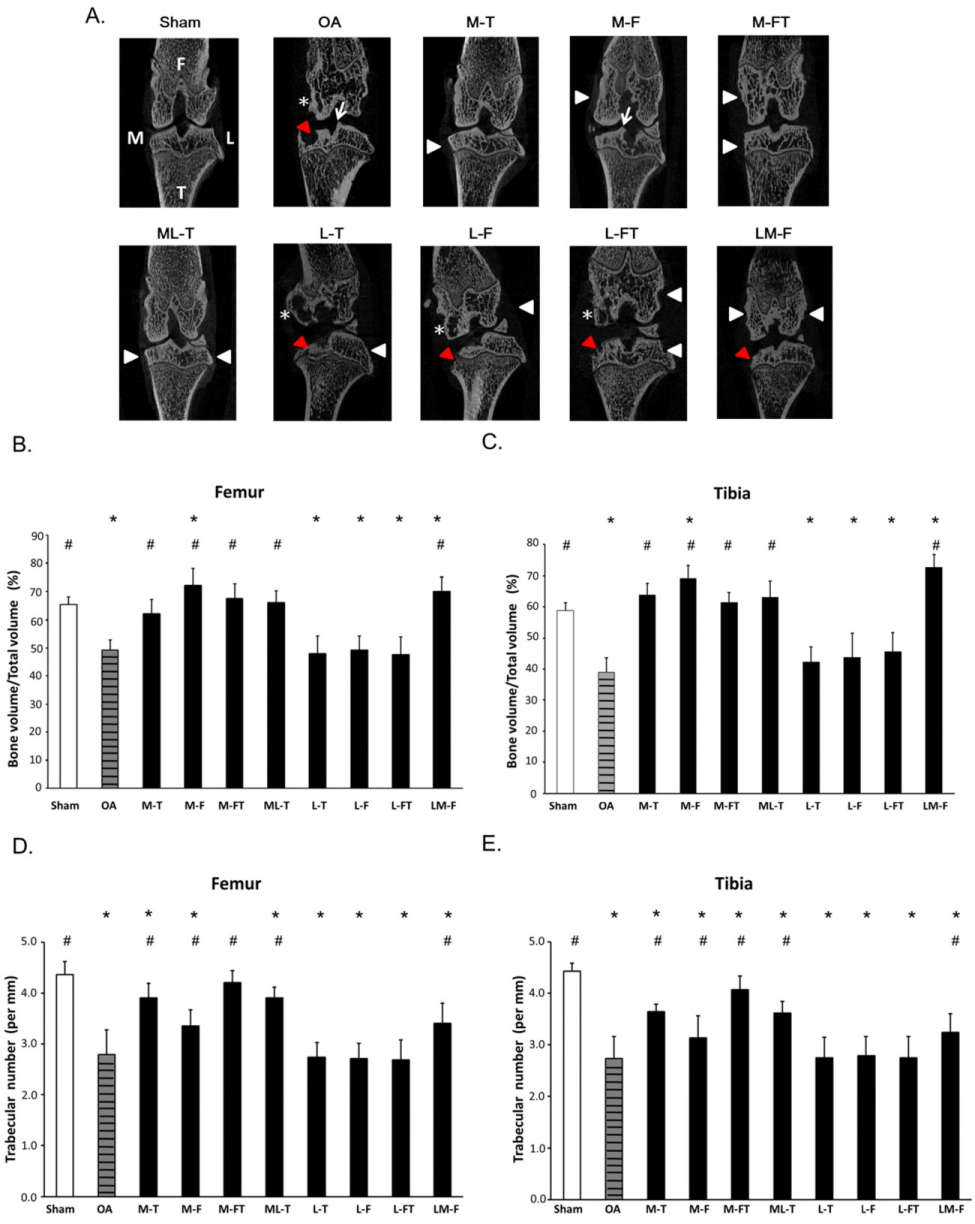
Micro-CT analysis. (A) Micro-CT images illustrated the positions of ESWT applied to the knee (white arrowheads) and the frontal-coronal view of the femur (F) and tibia (T) in each group by using CT-analyzer software (www.blue-scientific.com/bruker-ctan-micro-ct-software/). The anatomical side of the left knee OA joint was noted as medial (M). Osteophytes (white arrows), cysts (white star) and erosions (red arrowheads) formation were observed. The micro-CT data of the trabecular bone volume, and trabecular number in the subchondral bone of femur (B) (D) and tibia (C) (E) of the rat knee. ^*^P < 0.05 was as compared with Sham group. ^#^P < 0.05 was as compared with OA group. The lateral site is indicated by L. M-T as the medial tibia condyles. M-F as the medial femur condyle. M-FT as the medial femur and tibia condyle. ML-T as the medial and lateral tibia condyle. L-T as the lateral tibia condyle. L-F as the lateral femur condyle. L-FT as the lateral femur and tibia condyle. LM-F as the lateral and medial femur condyle.

For ESWT treatment, the rats were sedated with a 1:1 volume mixture of Rompun (5 mg/kg) (Bayer, Leverkusen, Germany) and Zoletil (20 mg/kg) (Virbac, Carros, France) in preparation. Ultrasound guidance (Toshiba Medical Systems Corporation, Tokyo, Japan) was employed to identify the precise anatomical locations for shockwave application. The source of the shockwave was an OssaTron (Saunwave, Alpharetta, GA, USA) and the impulse energy was focused on a focal point at the front of the balloon with the cross of a laser beam as guidance 29, 30. After pinpointing the precise locations for the treatment of OA of the knee, 800 impulses of shockwave at 14 kV (equivalent to 0.25 mJ/mm2) was applied in a single section to the specific sites as indicating in the Fig. 1 (white arrowheads) post-one week surgery.

### Experimental design

The Sprague-Dawley rats (n=100) were divided into ten groups (n=10 for group) as displayed in Fig. 1 and Supplemental Table 1. Group 1: Designated Sham. Rats received sham arthrotomy of the knees without inducing OA. Group 2: Designated OA. Rats underwent induction of knee OA only. Group 3: Designated M-T. Rats with knee OA plus ESWT at the medial tibia condyles post-surgery. Group 4: Designated M-F. Rats with knee OA plus ESWT at the medial femur condyles post-surgery. Group 5: Designated M-FT. Rats with knee OA plus ESWT at the medial femur and tibia condyles post-surgery. Group 6: Designated ML-T. Rats with knee OA plus ESWT at the medial and lateral tibia condyles post-surgery. Group 7: Designated L-T. Rats with knee OA plus ESWT at the lateral tibia condyles post-surgery. Group 8: Designated L-F. Rats with knee OA plus ESWT at the lateral femur condyles post-surgery. Group 9: Designated L-FT. Rats with knee OA plus ESWT at the lateral femur and tibia condyles post-surgery. Group 10: Designated LM-F. Rats with OA knee plus ESWT applied to the lateral and medial femur condyles post-surgery. During the 12 weeks post-treatment, some rats died without infection and might be due to the aggressive behaviors by attacking other weaker cage mates. Therefore, the rats were housed individually with providing the necessary environmental enrichments (1-2 days) and the data were analyzed from 8 to 10 rats per group after experiments. Finally, the rats (n=87) were sacrificed and the left knees were collected for further analysis (Fig. 1 and Table 1).

### Micro-CT scan

The rats were sacrificed at 12 weeks post-treatment (total 13 weeks post-surgery) and the knees of all groups were fixed with 4 % PBS-buffered formaldehyde at 4°C for 2 days and then were scanned using a micro-CT scanner (Skyscan 1076; Skyscan, Luxembourg, Belgium) with a filter A1 0.5 mm, exposure 270 ms, isotopic pixel size 18 × 18 × 18 μm, X-ray voltage 50 Kv and 500 μA. The trabecular volume, and trabecular number were calculated and computer analyzed from subchondral bone of femur and tibia. Image reconstruction was performed using Bruker SkyScan NRecon 2.0. (Bruker, Luxembourg, Belgium) and a series of planar transverse gray images was generated using CT-analyzer software (Skyscan; www.blue-scientific.com/bruker-ctan-micro-ct-software/).

### Safranin-O staining, osteoarthritis scoring and rating

**Figure 2:**
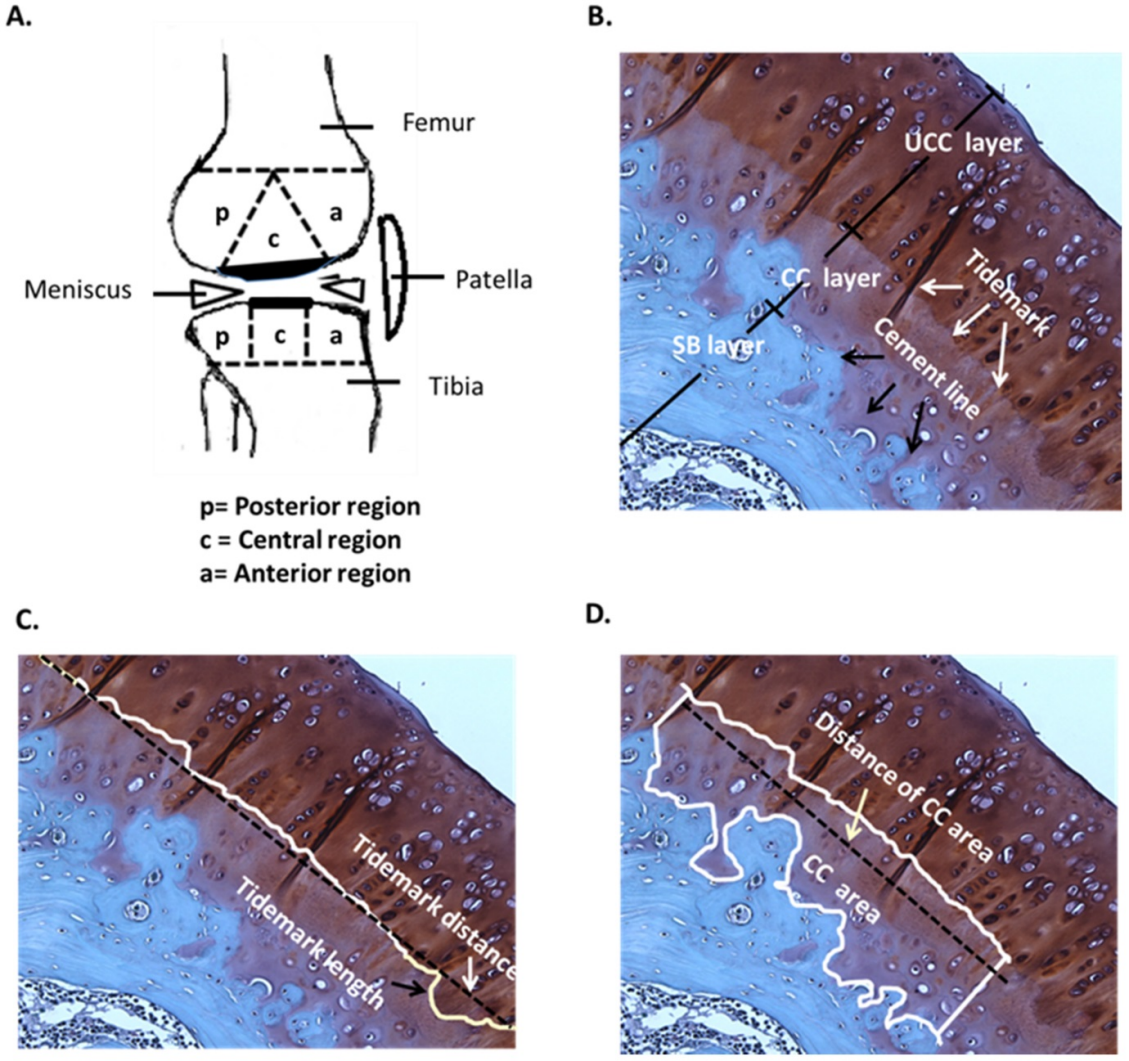
Pathological analysis. (A) Knee sketch displays the 3 sub-regions, which are the anterior (a), central (c) and posterior (p) regions, on the femoral and tibial condyles in the sagittal plane. (B) A section of the hyaline cartilage in the sagittal plane is stained with safranin-O, where the layers of the uncalcified cartilage (UCC; dark red), calcified cartilage (CC; light red) and subchondral bone (SB; blue) are shown. The tidemark (white arrows) is located between the UCC and CC layers, and the cement line (black arrows) is located between the CC and SB layers. The magnification is 400×. (C) The tidemark length (black arrow) is the actual length of the tidemark, and the tidemark distance (white arrow) is the shortest length of the tidemark. The magnification is 400×. (D) The CC area (white box) is the region between the UCC and SB layers, and the distance of the CC area is the length of the CC area (white arrow). The magnification is 400×. n = 8 to 10 for each group.

The rat knees were subjected to histopathologic examination after sacrifice and were cut with a bone chisel. All specimens were decalcified in EDTA at 37°C for approximately 1 month until the bones had softened, then embedded in paraffin blocks. Each block was cut using an automatic microtome (Leica, Wetzlar, Germany) and the sections were stained with safranin-O under the lab's protocol of Weight's iron hematoxylin solution for 10 min, 0.05% aqueous Fast-green for 5 min and 8% safranin-O for 5 min. Anatomically, the central regions of articular cartilage on femur and tibia usually received the greatest load-bearing forces during OA, and therefore, the evaluations of modified Mankin score system (scale range, 0 to 33) for osteoarthritis scoring and rating were mainly focused at the regions (Fig. 2). The score system assessed the factors of cartilage surface damage (0-6), loss of cellularity (0-3), loss of matrix staining (0-4), loss of tidemark integrity (0-1) and category of proportions of lesion site scoring (0-19) 31.

In addition, the level of knee OA was assessed according to the percentage of cartilage involvement in the articular surface of the knee and referenced by OA staging assessment using the OARSI scoring system as follows: Stage I, <10% cartilage involvement, classified as normal/minor; Stage II, 10% to 25% cartilage involvement, classified as mild; Stage III, 25% to 50% cartilage involvement, classified as moderate; and Stage IV, >50% cartilage involvement, classified as severe 32. Determination of the superficial OA area of the weight-bearing region of the femur or tibia was performed using NIH ImageJ (64-bit Fiji version; www/ImageJ.net/Fiji/Downloads) and ImagePro software (Media Cybernetics Inc., Rockville, MD, USA; www.mediacy.com). Therefore, the percentage of cartilage involvement was defined as cartilage involvement (%) = (OA surface area / Surface area of medial compartment) x 100% (Supplemental Fig. 1). In addition, the evaluations were blinked to the ID of groups, and both the stage and classification of the femur and tibia were determined according to the percentage of cartilage involvement values (Table 1).

### Determination of tidemark roughness and calcified cartilage thickness

In the histological analysis, the uncalcified cartilage (UCC) layer, CC layer and subchondral bone (SB) layer, tidemark (white arrow) and cement line (black arrow) were all indicated by safranin-O staining (Fig. 2A and 2B). The mean of tidemark roughness (R_tm_) was defined as R_tm_ = (Tidemark length / Tidemark distance), where tidemark length was the actual length of the tidemark and tidemark distance was the shortest length of the same tidemark (Fig. 2C). Therefore, a higher R_tm_ ratio indicated a wavier tidemark, and R_tm_=1.00 was defined as no tidemark roughness, which is usually caused by severe erosion and interruption and is considered non-definable 33. The mean of the CC thickness (CC_thick_) was defined as CC_thick_ = (CC area / Distance of CC area), where the distance of the CC area was the shortest length of a specific CC area (Fig. 2D). All calculations were based on the mathematical definitions and formulas described 33. Safranin-O staining was imaged using a charge-coupled camera. The structure and pattern distributions of the tidemark and CC area were discussed and carefully determined by the research team, in order that the length of the tidemark and thickness of the CC were accurately traced and automatically determined using NIH ImageJ (64-bit Fiji version; www/ImageJ.net/Fiji/Downloads) and ImagePro software (www.mediacy.com).

### Statistical analysis

SPSS version 26.0 (SPSS Inc., Chicago, IL, USA; www.ibm.com/tw-zh/analytics/spss-trials) was used for statistical analysis. Calculated data are expressed as the mean ± SD, and one-way analysis of variance (ANOVA) with *post hoc* Tukey tests (normal distribution) were used for group comparisons of R_tm_ and CC_thick_. Ranking data, such as the modified Mankin score, were expressed as the mean ± SEM, and the Kruskal-Wallis test was employed for multiple non-distributional comparisons. Pearson's correlation coefficient *r* was used for simple correlation analysis to examine the correlations of the modified Mankin score with R_tm_ and CC_thick_, of CC_thick_ with R_tm_, and of the Euclidean distance with the percentage of cartilage involvement 34. The levels of significance were set at *P* < 0.05. For the three‐dimensional scatter plots, the scale variable ranges were selected according to the minimum and maximum values obtained from the actual measurements of the modified Mankin score (x-axis), R_tm_ (y-axis) and CC_thick_ (z-axis), consistently normalized to be 0% to 100% for convenience of group distinction and interpretation. The graphs were created using SigmaPlot 12.0 (Systat Software, Inc, CA, USA). Therefore, a coordinate consisted of the modified Mankin score, R_tm_ and CC_thick_ in the 3-D scatter plot. The physical connection distance between two points was estimated as the Euclidean distance:


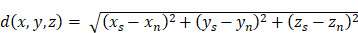

(1)

where x_s_, y_s_ and z_s_ were the coordinates of a specific point, and x_n_, y_n_ and z_n_ were the mean coordinates of the Sham group (2.00, 77.647, 26.456 in the femur; 3.00, 85.739, 37.950 in the tibia), with the unit designated as an arbitrary unit (a.u.). Principle component analysis (PCA) biplots were generated and the variables consisted of the modified Mankin score, R**_tm_**, CC**_thick_**, Euclidean distance, and surface cartilage involvement by using Sigma blot version 14.0 (System Software, Inc., CA, USA; www.systatsoftware.com/products/sigmaplot/).

## Results

### Micro-CT showed recovery of subchondral bone after ESWT in knee OA

The frontal micro-CT images illustrated the positions of ESWT application (arrow heads) and the morphology of the subchondral bone after treatment (Fig. 1A). In brief, the Sham group revealed subchondral plate integrity with smooth contours. The OA, L-T, L-F and L-FT groups displayed severe erosion and pitting on the weight-bearing areas of the subchondral plate and the formation of notable cysts (star). The M-T, M-FT and ML-T groups revealed no obvious erosion, trabecular bone thickening or sclerosis in the subchondral bone. The M-F and LM-F groups exhibited severe superficial sclerosis and osteophyte formation (arrow) in the subchondral bone of the medial compartment.

In the micro-CT analysis, ESWT significantly increased the percentage of trabecular bone volume (BV/TV) and trabecular number in medial groups (M-T, M-F, M-FT and ML-T ) than lateral groups (L-T, L-F and L-FT) of subchondral bone of femur (Figure 1B and 1D) and tibia (Figure 1C and 1E) by compared with Sham (P < 0.05) and OA group (P < 0.05).

### Clarification of the articular cartilage features by morphometric analysis in knee OA after ESWT

In order to assess the changes in tissue composition, in-depth analysis of the histomorphometric articular cartilage of the femur and tibia was performed as described in Materials and Methods, including calculation of the modified Mankin score, and assessment of R**_tm_** and CC_thick_ by Safranin-O staining at central regions of the femur and tibia in each group (Fig. 2A to 2D).

Typically, the articular cartilage of the Sham group presented a smooth surface with no structural damage, a high UCC thickness and a low CC_thick_, as shown in Fig. 2. The hyaline cartilage in the M-T, M-FT and ML-T groups revealed integrity and a smooth cartilage surface, as well as relatively normal chondrocytic morphologies and arrangements. Further, the M-T and ML-T groups exhibited higher CC_thick_ in the femur and tibia than the M-FT group. The L-T, L-F and L-FT groups exhibited poor efficacies of treatment, as seen in the OA group, presenting as erosion beyond the tidemark, few chondrocytes, many hypertrophic chondrocyte clusters, and frequent interruption of tidemark intactness, with indistinguishable UCC and CC layers. Notably, the M-F and LM-F groups presented with severe tissue damage, including fibrosis on the surface layer, loss of extracellular matrix, the presence of hypertrophic chondrocytes in a cluster arrangement, and CC thickening.

### Classification of OA severity using the OARSI scoring system

**Figure 3:**
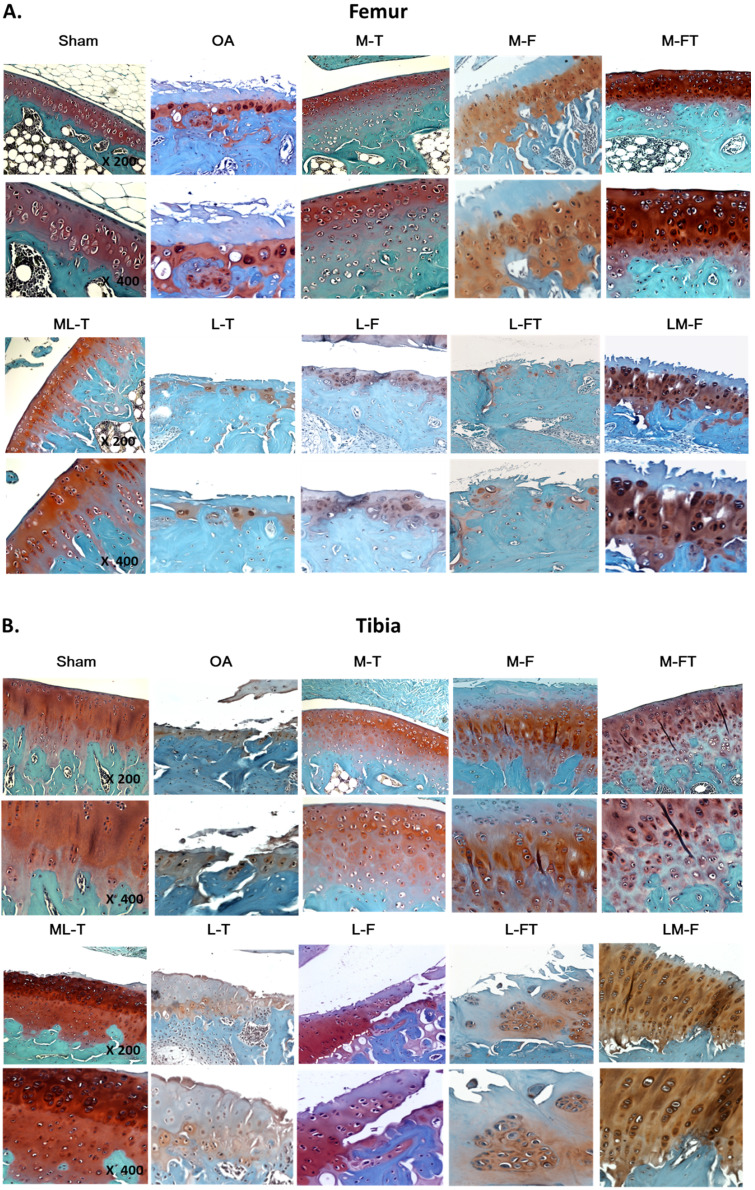
Cartilage matrix conditions of the femur and tibia following ESWT application to various sites. The femoral (A) and tibial (B) condyle sections of the articular osteochondral tissues are stained with safranin-O and observed at 200× magnification and 400× magnification. n = 8 to10 for each group. M-F as the medial femur condyle. M-FT as the medial femur and tibia condyle. ML-T as the medial and lateral tibia condyle. L-T as the lateral tibia condyle. L-F as the lateral femur condyle. L-FT as the lateral femur and tibia condyle. LM-F as the lateral and medial femur condyle.

Stage classification of OA severity was performed using the OARSI scoring system. The percentage of surface damage caused by OA and the percentage of surface cartilage involvement following ESWT at different positions of the knee are shown in Fig. 3 and Table 1.

In the femur, the Sham group exhibited the lowest surface cartilage involvement, at 0.606 ± 0.441%, which was at Stage I and classified as normal. The M-FT, ML-T and M-T groups exhibited surface cartilage involvement of 13.321 ± 5.654%, 20.392 ± 7.915% and 23.679 ± 9.252%, respectively, which were at Stage II and classified as mild. The M-F and LM-F groups exhibited surface cartilage involvement of 48.012 ± 11.808% and 49.274 ± 12.797%, respectively, which were at Stage III, and classified as moderate. The L-T, OA, L-FT and L-F groups exhibited surface cartilage involvement of 89.197 ± 10.195%, 89.233 ± 12.932%, 91.562 ± 8.183% and 91.924 ± 13.859%, respectively, which were at Stage IV, and classified as severe.

In the tibia, the Sham group (0.258 ± 0.647%) had the lowest surface cartilage involvement, which was at Stage I, and classified as normal. The M-FT, M-T and ML-T groups exhibited surface cartilage involvement of 18.643 ± 7.597%, 23.263 ± 6.537% and 24.281 ± 7.304%, respectively, which were at Stage II, and classified as mild. Further, the surface cartilage involvement in the LM-F group was 48.464 ± 9.149%, which was at Stage III, and classified as moderate. Finally, the surface cartilage involvement in the M-F, L-FT, OA, L-F and L-T groups was 53.147 ± 7.340%, 83.008 ± 15.806%, 86.396 ± 9.874%, 86.507 ± 8.809% and 87.253 ± 12.649%, respectively, which were at Stage IV, and classified as severe. The results clearly showed differences in the level of tissue recovery after ESWT at different positions of the knee for the treatment of OA.

### Correlation of the modified Mankin score with R_tm_

**Figure 4:**
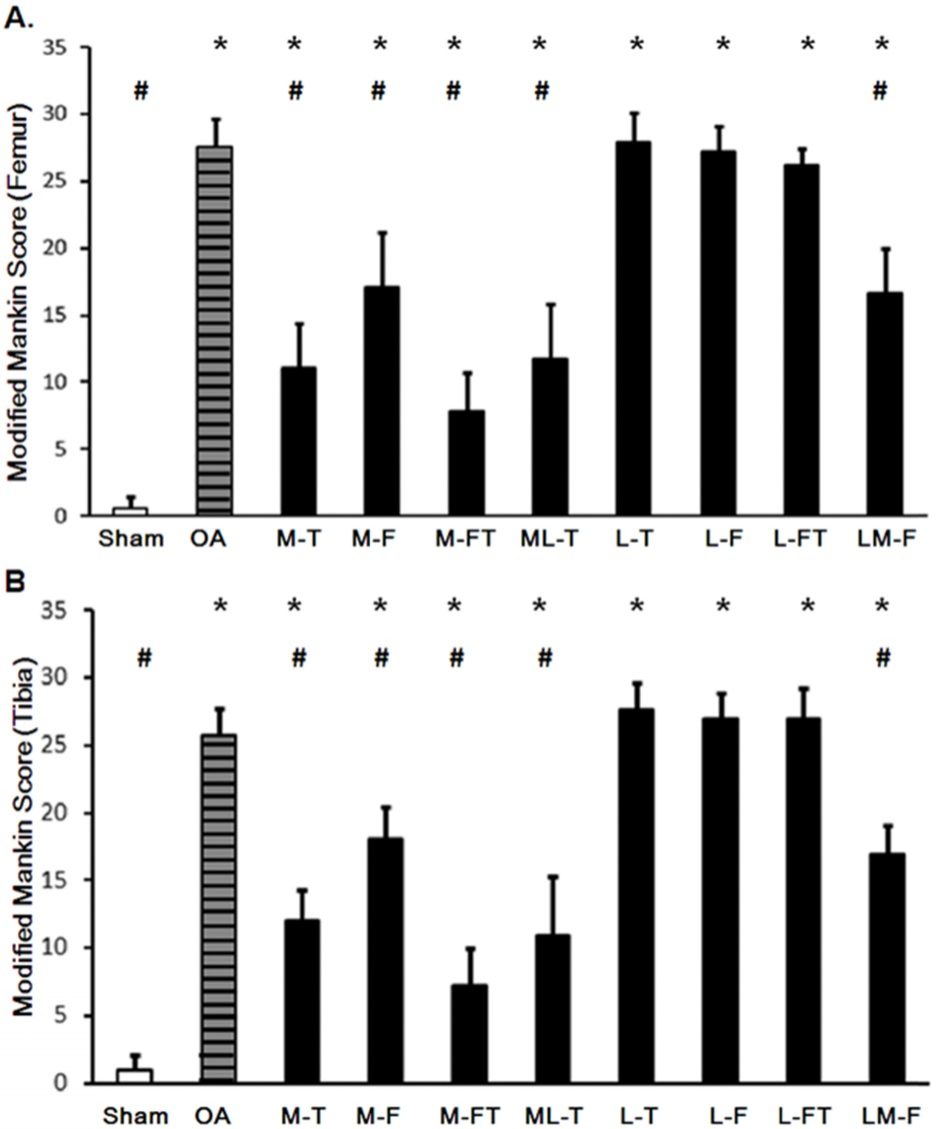
Changes in the articular cartilage evaluated by the modified Mankin score after ESWT at various sites of the femur (A) and tibia (B). * indicates the difference as compared with the Sham group, P < 0.05; # indicates the difference as compared with the OA group; P < 0.05. n = 8 to 10 for each group. M-F as the medial femur condyle. M-FT as the medial femur and tibia condyle. ML-T as the medial and lateral tibia condyle. L-T as the lateral tibia condyle. L-F as the lateral femur condyle. L-FT as the lateral femur and tibia condyle. LM-F as the lateral and medial femur condyle.

The application of ESWT at different positions resulted in various levels of improvement in the articular cartilage in knee OA (Fig. 3 and Supplemental Table 2). The modified Mankin scores of the experimental groups revealed significant recovery of the cartilage in the central regions of the femur and tibia after ESWT (Fig. 3 and Fig. 4). The modified Mankin score results presented in Fig. 4A and 4B indicated that the outcomes in the medial groups (M-T, M-F, M-FT and ML-T) were generally better than those in the lateral groups (L-T, L-F, L-FT and LM-F) as compared with the corresponding locations in the Sham (P < 0.05) and OA groups (P < 0.05). Further, the M-FT group had the best healing score among the medial groups following treatment for OA (P < 0.05). There were, however, no statistical differences in the modified Mankin score in the L-T, L-F and L-FT groups as compared with the OA group.

**Figure 5:**
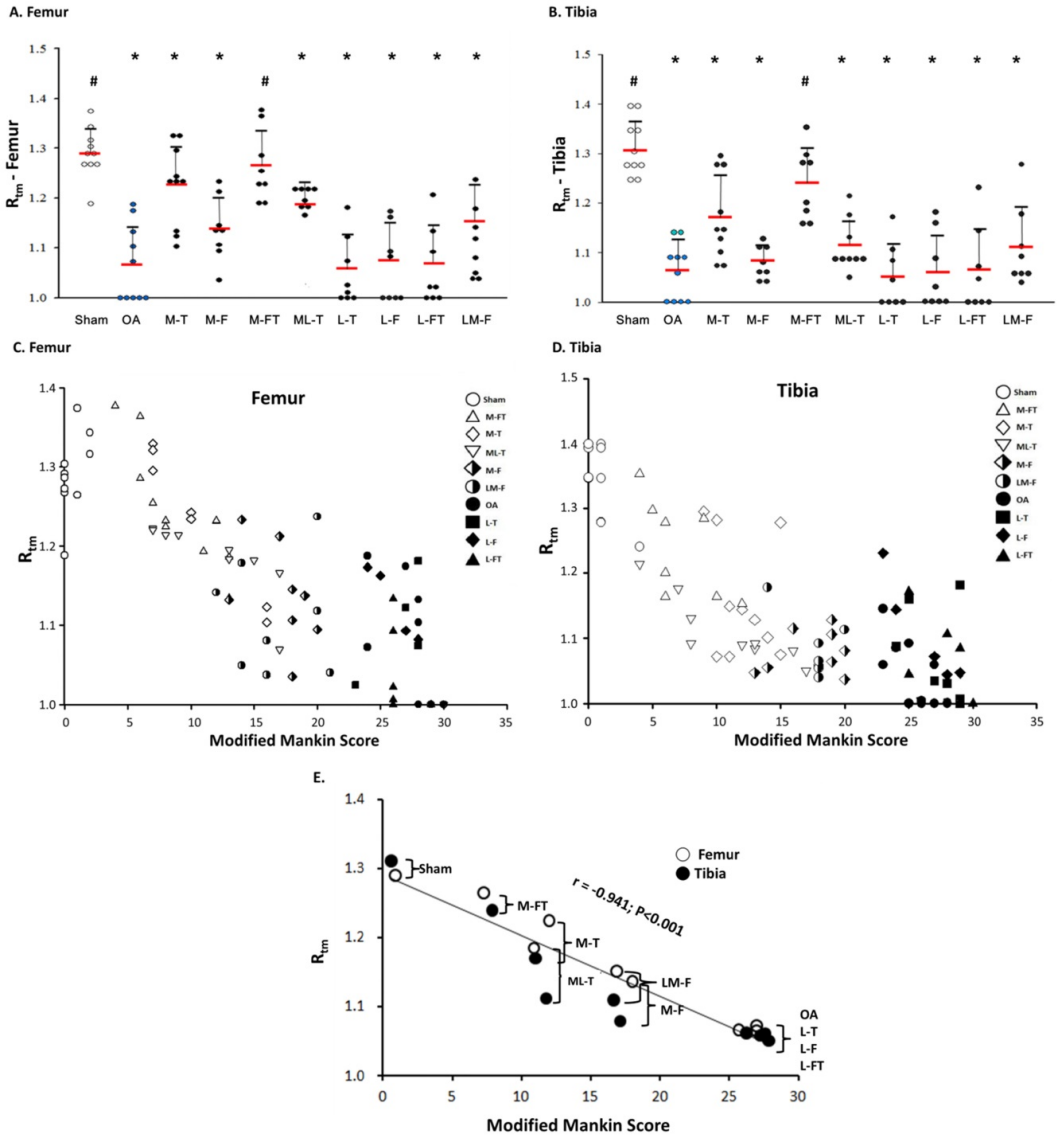
Tidemark roughness (R_tm_) in the central regions of the femur (A) and tibia (B) condyles. * indicates the difference as compared with the Sham group, P < 0.05; # indicates the difference as compared with the OA group, P < 0.05. Undetermined Rtm is indicated as a score of 1. n = 8 to 10 for each group. The correlation of all groups between the modified Mankin score and R_tm_ are presented in the femur (C) and tibia (D) after ESWT. (E) The mean correlation between the modified Mankin score and R_tm_ was negative, with r = -0.941; P < 0.001. M-F as the medial femur condyle. M-FT as the medial femur and tibia condyle. ML-T as the medial and lateral tibia condyle. L-T as the lateral tibia condyle. L-F as the lateral femur condyle. L-FT as the lateral femur and tibia condyle. LM-F as the lateral and medial femur condyle.

Next, the R_tm_ was analyzed, and only in the M-FT group was there a statistically significant difference as compared with the Sham group among the ESWT groups in the femur and tibia (Fig. 5A and 5B; Supplemental Table 3). In addition, the Sham and M-FT groups displayed statistically significant differences in the R_tm_ as compared with the OA group (P < 0.05). The results indicated that the M-FT group had a higher R_tm_ after ESWT for the treatment of knee OA than the other groups in the femur and tibia.

Finally, the correlations between the each group and the means of the modified Mankin score and R_tm_ in all groups for the femur and tibia are illustrated in Figure 5C, 5D and 5E. The results of means of correlations which was clearly showed a very strong negative correlation between the modified Mankin score and R_tm_ in the Sham, OA, and all treatment groups (Figure 5E) (r = -0.941; P < 0.001). Further, the co-ordinates of the Sham group had the lowest modified Mankin scores and the highest R_tm_ values (Fig. 5E). In contrast, the OA, L-T, L-F and L-FT groups presented the highest modified Mankin scores and lowest R_tm_ values.

### Relationships between modified Mankin score, CC_thick_ and R_tm_ after ESWT

**Figure 6:**
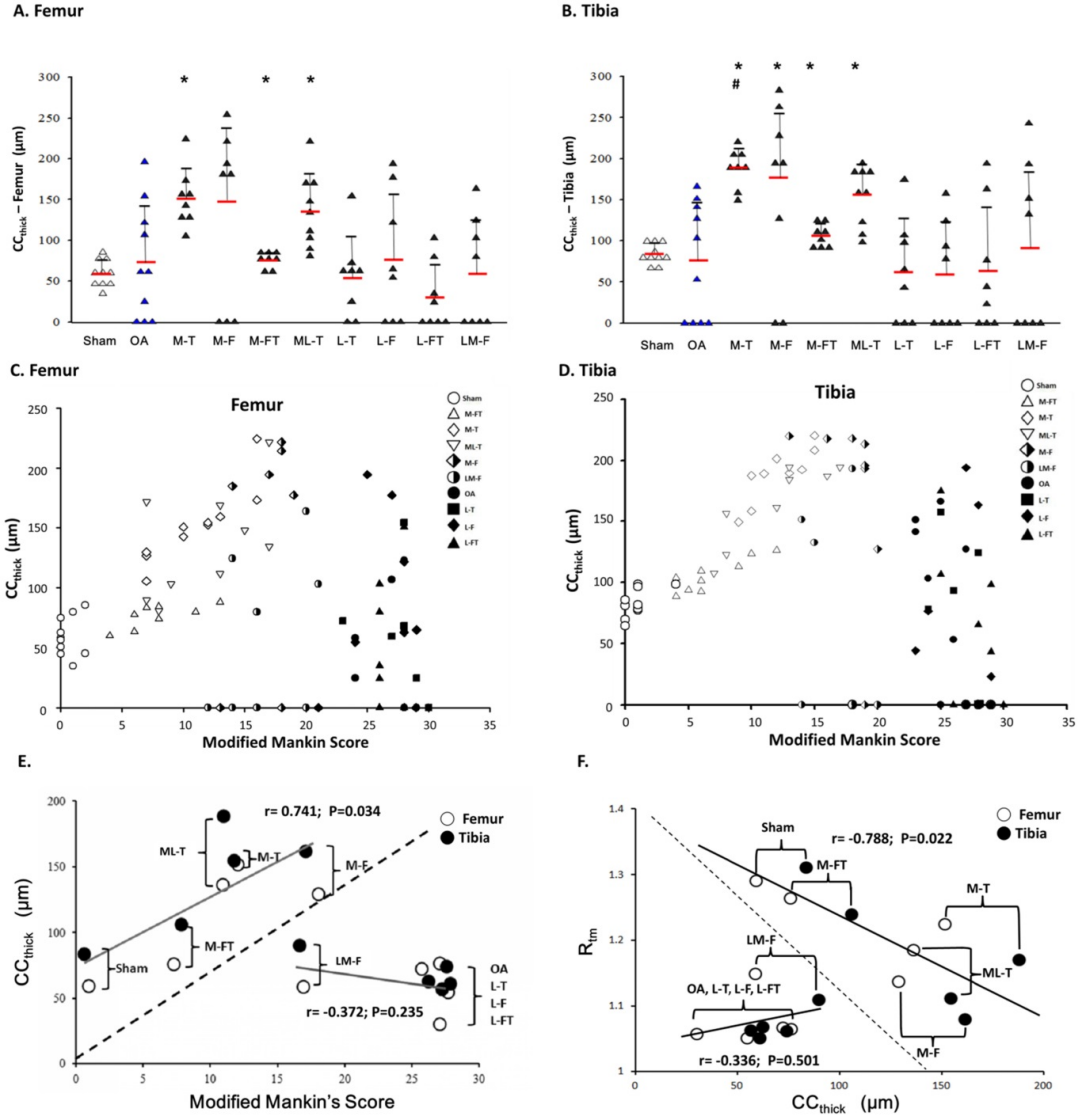
Calcified cartilage thickness (CC_thick_) in the central femur (A) and tibia (B) condyles. There were no significant differences when comparing the OA group with the other groups due to the high degree of fluctuation in standard deviation. * indicates the difference as compared with the Sham group, P < 0.05; # indicates the difference as compared with the OA group, P < 0.05. n = 8 to 10 for each group. The correlation of all groups between the modified Mankin score and CC_thick_ are presented in the femur (C) and tibia (D) after ESWT. (E) The mean correlation between the modified Mankin score and CC_thick_ was significant (r = 0.746; P = 0.034) in the Sham, M-FT, ML-T, M-T and M-F groups; however, there was no significant correlation (r = -0.372; P = 0.235) in the LM-F, OA, L-T, L-F and L-FT groups. (F) The mean correlation between the R_tm_ and CC_thick_ was significant (r = -0.788; P = 0.022) in the Sham, M-FT, ML-T, M-T and M-F groups; however, there was no significant correlation (r = 0.336; P = 0.501) in the LM-F, OA, L-T, L-F and L-FT groups. M-F as the medial femur condyle. M-FT as the medial femur and tibia condyle. ML-T as the medial and lateral tibia condyle. L-T as the lateral tibia condyle. L-F as the lateral femur condyle. L-FT as the lateral femur and tibia condyle. LM-F as the lateral and medial femur condyle.

In the analysis of the femur, the M-T, M-FT and ML-T groups exhibited significant differences as compared with the Sham group (P < 0.05) (Fig. 6A). There were no significant differences in CC_thick_ between any of the groups in comparison with the OA group due to the high fluctuation in standard variation (Fig. 6A and Supplemental Table 4). On the other hand, in the analysis of the tibia, significant differences in CC_thick_ were observed in the M-T, M-F, M-FT and ML-T groups as compared with the Sham group (P < 0.05). Further, only the M-T group exhibited a significant difference as compared with the OA group (Fig. 6B and Supplemental Table 4) (P < 0.05). The correlations of the modified Mankin score and CC_thick_ in each group was displayed in femur and tibia after ESWT (Figure 6C and 6D). Further, there was no statistically significant correlation between the mean of the modified Mankin score and CC_thick_ in all the groups of the femur and tibia after ESWT (Fig. 6C and 6D). The correlations between the means of R_tm_ and CC_ thick_ in all groups are clearly presented in Figure 6E. The positive correlations (r=0.756, P=0.034) were found among the mean of the modified Mankin score and CC_thick_ in Sham, M-T, M-F, M-FT and ML-T groups (Figure 6E). In addition, the OA, L-T, L-F, L-FT and LM-F groups exhibited a negative correlation trend without significance (r = -0.372, P = 0.235). It was observed that the results of the Sham, M-T, M-F, M-FT and ML-T groups were significantly negatively correlated (r= -0.788; P=0.022), and the OA, L-T, L-F, L-FT and LM-F groups exhibited positive trends without significant correlation (r = 0.336, P = 0.501).

### Pathological and Morphometric correlations of the modified Mankin score, R_tm_ and CC_ thick_ in the femur and tibia

**Figure 7:**
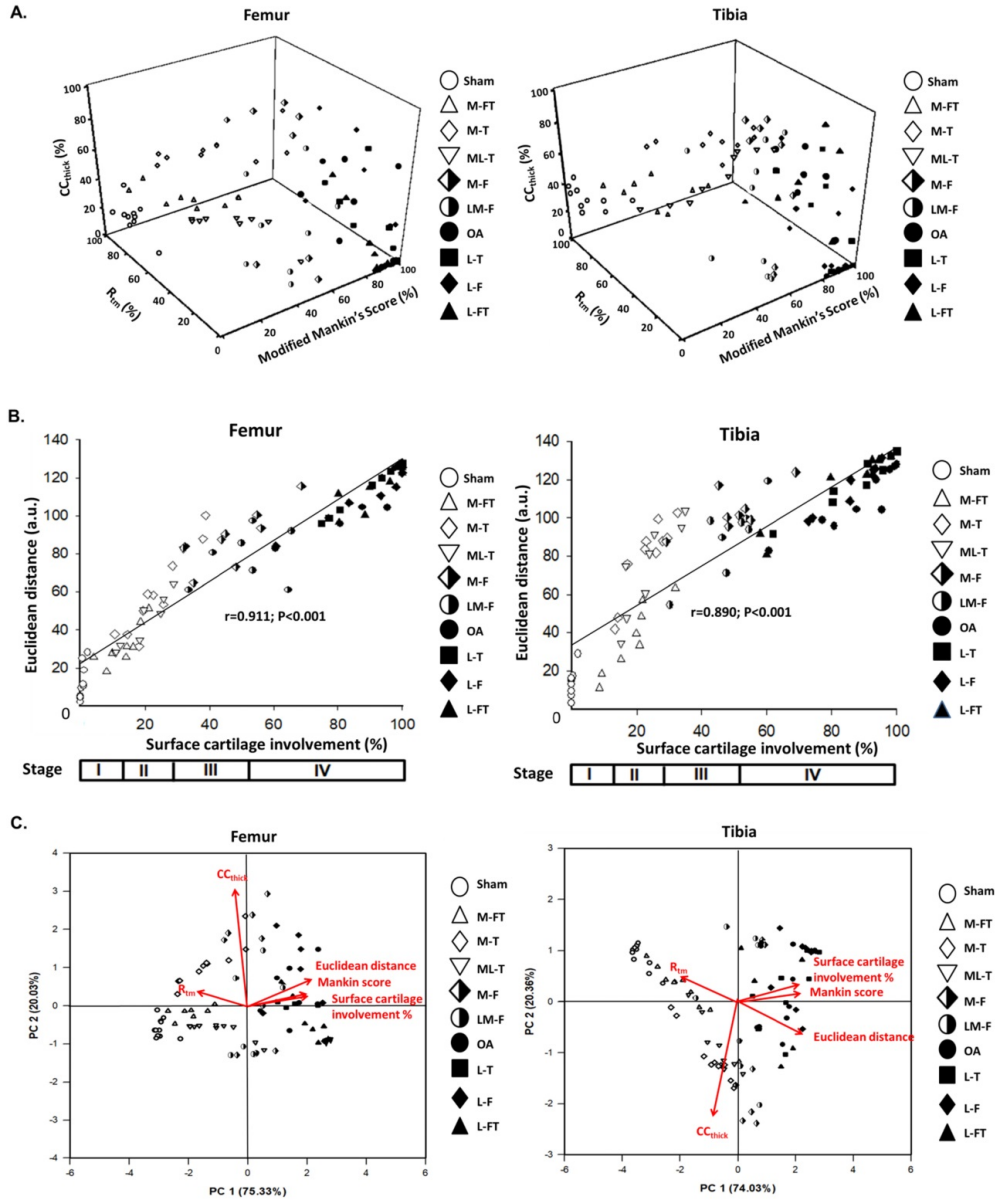
Pathological and Morphometric correlations. (A) Correlations of three‐dimensional scatter plots with (B) Euclidean distance and percentage of cartilage involvement for the femur and tibia as assessed from the data of the modified Mankin score, CC_thick_, and R_tm_ after ESWT at different positions of the rat OA knee. (C) Principle component analysis (PCA) of the femur and tibia with multiple inter-correlated variables including modified Mankin score, CC_thick_, R_tm_, Euclidean distance, and surface cartilage involvement by using Sigma blot version 14.0 (https://systatsoftware.com/products/sigmaplot/). n = 8 to 10 for each group. M-F as the medial femur condyle. M-FT as the medial femur and tibia condyle. ML-T as the medial and lateral tibia condyle. L-T as the lateral tibia condyle. L-F as the lateral femur condyle. L-FT as the lateral femur and tibia condyle. LM-F as the lateral and medial femur condyle.

In order to further elucidate the correlations of the modified Mankin score with R_tm_ and CC_thick_ in the femur and tibia after ESWT at different locations of the knee in OA, three‐dimensional scatter plots were created for all groups, as shown in Fig. 7A. The results showed the general distributions of the Sham, M-FT, M-T and ML-T groups (blank symbols), which had lower percentages of the modified Mankin score and CC_thick_, but higher percentages of R_tm_; hence, they were scattered on the left dimension. In contrast, the distributions of the OA, L-T, L-F and L-FT groups (filled symbols) were scattered on the right dimension with higher percentages of the modified Mankin score and CC_thick_, but lower percentages of R_tm_. In addition, the M-F and LM-F groups (half-filled symbols) overlapped the two aforementioned distributions. The Euclidean distances of all samples were then determined, and the correlation between Euclidean distance and surface cartilage involvement was strongly positive in the femur (r = 0.911, P < 0.001) and tibia (r = 0.890, P < 0.001) (Fig. 7B). In brief, the M-FT, ML-T and M-T groups had lower Euclidean distances and surface cartilage involvement (Stage II); the M-F and LM-F groups had medium Euclidean distances and surface cartilage involvement (Stage III); and the OA, L-T, L-F and L-FT groups had high Euclidean distances and surface cartilage involvement (Stage IV).

### Principle component analysis (PCA) of ESWT at various locations in the femur and tibia

The results of principle component analysis (PCA) in the femur and tibia enabled ranking of the ESWT effectiveness among the groups, as assessed from multiple inter-correlated variables (Fig. 7C). The biplots of the femur and tibia showed that the grouping patterns for the OA and lateral groups (L-T, L-F and L-FT) were clearly different from those of the Sham and medial groups (M-FT, M-T and ML-T). Furthermore, the OA and lateral groups were characterized by positive correlations of sufrace cartilage involvement, modified Mankin score and Euclidean distance, and negative correlations of R_tm_, which suggested that the lateral groups had a higher OA severity. In contrast, the Sham and medial groups displayed a positive correlation of R_tm_, but negative correlations of surface cartilage involvement, modified Mankin score and Euclidean distance. These results suggested that the medial groups had a lower OA severity. Notably, the plots of the M-F and LM-F groups showed scattering along the origin of the loading vectors, which were plotted between the medial and lateral groups. Moreover, CC_thick_ was identified as the most important factor in the treatment of knee OA by ESWT.

## Discussion

In this study, we first observed and measured the pathological changes and correlations of the modified Mankin score, R_tm_ and CC_thick_ by statistical methods after ESWT at different positions of the knee for the treatment of OA. Furthermore, detailed examination of the efficacy of treatment for each of the treatment positions was performed by examination of 3D scatter plots (modified Mankin score, R_tm_ and CC_thick_), Euclidean distance, percentage of cartilage involvement and PCA. Our study results further elucidated the degree of the osteochondroprotective characteristic of ESWT in the treatment of knee OA at various positions.

The tidemark was obvious in the deep zone of the articular cartilage, which is between the hyaline cartilage and the CC (Fig. 2B) 32, 35. The structure of the tidemark is correlated with the articular loading, and is a result of the accumulation of mineralized factors such as calcium to form dense and dark lines 36. The R_tm_ changes according to the mechanical loading force and is significantly reduced in OA or PTOA 17. ESWT has been reported to have chondroprotective effects; however, the change in the tidemark after treatment remains unclear 23, 24. This study examined the change in R_tm_ after ESWT, and found it to be location-dependent, and the recovery of tissues was very similar to that in the Sham group. These results suggested that application of ESWT at the appropriate location had the ability to promote tissue repair in the structure of the hyaline cartilage after damage to the knee caused by OA.

The results of PCA showed that the CC_thick_ was a key factor after ESWT for the treatment of knee OA (Fig. 7C). In fact, the CC_thick_ plays a significant role in transmitting mechanical stress, nutrition and biological stimuli between the hyaline articular cartilage and the subchondral bone 37, 38. It can suffer a mechanical force up to 15 MPa, while that of the subchondral bone is around 4 GPa 39, 40. When trauma to a joint occurs, CC_thick_ injuries are observed before injuries to the subchondral bone. Researchers have reported that thickening of the CC is observed, leading to advancement of CC_thick_ during the OA process 41. However, the CC layers disappear or are undetectable in severe OA, or following failure of ESWT at various positions, owing to a high degree of tear and wear, leading to severe abrasion and erosion of the CC layers (Fig. 3). In this study, we observed that most medial positions of ESWT application to the knee, such as in the M-FT, M-T and ML-T groups, resulted in recovery of the morphology of the CC, and therefore it could be considered that ESWT at the medial positions could somehow trigger a stronger osteochondral-protective effect than lateral positions, such as in the L-T, L-F and L-FT groups. Interestingly, the M-F and ML-F groups had mixed high and low CC_thick_, which suggested that ESWT at these positions might induce a less-stable osteochondral-protective effect than observed in the M-FT, M-T and ML-F groups. Therefore, relationships between the position of ESWT around the knee and the release and induction of repair and/or growth factors can be theorized. However, the mechanisms require further investigation.

PCA of the femur and tibia indicated that the success of ESWT was location-sensitive, and the therapeutic effectiveness was ranked as follows: **1)** the M-FT group, which exhibited best therapeutic result, with histomorphometric characteristics closest to those of the Sham group;** 2)** the M-T and M-FT groups, which were generally characterized by a high CC_thick_ and a low R_tm_; **3)** the M-F and LM-F groups, which possessed the characteristics of flexible changes in CC_thick_; **4)** the L-T, L-F and L-FT groups, which were characterized by a high percentage of OA involvement, a high modified Mankin score and a greater Euclidean distance, but a low R_tm_.

The principle of the clinical approach to PTOA is similar to that of the approach to primary OA of the knee. Nonsurgical treatments are the first-line therapy, including therapeutic and strengthening exercises, weight reduction, insole application, non-steroidal anti-inflammatory drugs and platelet-rich plasma (PRP) injection 42, 43. Patients in whom conservative treatment fails are often referred for surgical intervention, including arthroscopic debridement, and even total knee arthroplasty for advanced OA. For early OA of the knee, arthroscopic debridement is not a recommended procedure according to the latest research 43. Owing to its less-invasive nature, ESWT has been applied for the treatment of various musculoskeletal disorders 21, 22, 44, 45. As with traditional indications, the novel application of ESWT to the medial compartment in early OA knees has been reported to have a positive retardation effect, and even a regressive effect, in an animal model. Previous reports have mentioned that ESWT to the subchondral bone of the medial plateau is better than application to other sites of the knee 27, 28; however, the detailed pathological changes have not been discussed in depth. The results of the present study demonstrated histological changes in R_tm_ and the articular CC_thick_ induced by ESWT at different locations of the knee in the treatment of OA. Furthermore, the study indicated promising results for the future regarding the application of ESWT to the subchondral bone and at the junction of the subchondral bone and cartilage, which are feasible in clinical practice. According to the encouraging findings of this study, the precise position of ESWT to achieve the best outcome for the treatment of OA is clear according to the pathological evidence, and the treatment is simple to perform under radiological imaging guidance.

There were several limitations of our study, as follows. The results of a small animal model used to explain the therapeutic effects of ESWT may differ from those of a larger animal model or a human clinical trial. The dosage of ESWT was selected for animal studies; however, it may not be the optimal dose for human subjects. The changes in R_tm_ and the articular CC_thick_ after ESWT in this OA animal study indicated the possibility of histopathological changes in the human knee, but any clinical relevance or correlations of progressive cartilage changes in the human knee are as yet unknown. In this study, when the knees of animal received artificial induction of OA by ACL and medial menisectomy, they experienced the unbalance of mechanical loading stress by meniscal destabilization. Eventually, the medial tibia articular regions could develop severe coronal and trabecular mal-alignments, which were considered as the key factor for OA initiation 46-49. Therefore, we theorized that ESWT application on medial tibia subchondral bone in early OA stage could triggered the appropriate tissue repair factors, such as BMPs, Wnt and VEGF and that might eventually alleviated OA progression 50. Although the exact mechanism is not clear, our experimental results demonstrating that the medial knees generally display better chondroprotective effects than the lateral knees in response to ESWT.

## Conclusion

ESWT acts as a mechanical stimulus that promotes biological healing processes through mechanotransduction 21, 45. Biological effects of ESWT have been reported, such as tissue regeneration, wound-healing, angiogenesis, bone remodeling, and anti-inflammation 21, 44. In addition, the biophysical nature of ESWT for OA has been reported in many studies, such as the variation of therapeutic effectiveness with impulse number and energy intensity 24, 51. However, there are almost no published manuscripts that discuss the correlation of ESWT location with treatment effectiveness in the treatment of knee OA. To the best of our knowledge, we assessed for the first time topical ESWT application at up to ten different combinations of locations for the treatment of OA in order to study the degree of treatment effectiveness in a rat knee OA model. Our previous studies and the present study demonstrated that ESWT applied to the subchondral bone of the tibia and femur resulted in a better structural integrity than ESWT at other sites, in terms of cartilage thickness, chondrocytic survival, and trabecular thickness 27, 28. Most importantly, we discussed in detail the application of ESWT at different positions of the knee for the treatment of OA, and further elucidated why ESWT in the M-FT group was more successful than in the other groups by analysis of the pathological changes and examination of the correlations of the modified Mankin score, R_tm_ and CC_thick_, in addition to the use of statistical analysis methods.

## Supplementary Material

Supplementary tables.

## Figures and Tables

**Table 1:** Surface cartilage involvement, stage and classification of each group.

Femur										
Group Name	Sham	M-FT	ML-T	M-T	M-F	LM-F	L-T	OA	L-FT	L-F
Sample (n)^1^	10	8	9	10	8	8	8	10	8	8
OA invol. %^2^	0.606 ± 0.441	13.321 ± 5.654	20.392 ± 7.915	23.679 ± 9.252	48.012 ± 11.808	49.274 ± 12.797	89.197 ± 10.195	89.233 ± 12.932	91.562 ± 8.183	91.924 ± 13.859
Stage	I	II	II	II	III	III	IV	IV	IV	IV
Classification	normal	mild	mild	mild	moderate	moderate	severe	severe	severe	severe
**Tibia**										
Group Name	Sham	M-FT	M-T	ML-T	LM-F	M-F	L-FT	OA	L-F	L-T
Sample (n)	10	8	10	9	8	8	8	10	8	8
OA invol. %	0.258 ± 0.647	18.643 ± 7.597	23.263± 6.537	24.281 ± 7.304	48.464 ± 9.149	53.147 ± 7.340	83.008± 15.806	86.396 ± 9.874	86.507± 8.809	87.252± 12.649
Stage	I	II	II	II	III	IV	IV	IV	IV	IV
Classification	normal	mild	mild	mild	moderate	severe	severe	severe	severe	severe

1. Sample (n)= The amount of rats. 2. The surface cartilage involvement (OA invo. %)= Surface OA area / Total surface area of medial compartment x 100%. 3. M-F as the medial femur condyle. M-FT as the medial femur and tibia condyle. ML-T as the medial and lateral tibia condyle. L-T as the lateral tibia condyle. L-F as the lateral femur condyle. L-FT as the lateral femur and tibia condyle. LM-F as the lateral and medial femur condyle.
