# Supplementary material for: Pathological, Morphometric and Correlation Analysis of the Modified Mankin Score, Tidemark Roughness and Calcified Cartilage Thickness in Rat Knee Osteoarthritis after Extracorporeal Shockwave Therapy
Source: Int J Med Sci. 2022 Jan 1;19(2):242–56. doi: 10.7150/ijms.67741 (PMC8795810; doi:10.7150/ijms.67741)
Supplement: Supplementary file 1 — Supplementary tables. [file ijmsv19p0242s1.pdf]

Pathological, Morphometric and Correlation Analysis of the Modified Mankin Score, Tidemark Roughness and Calcified Cartilage Thickness in Rat Knee Osteoarthritis after Extracorporeal Shockwave Therapy

Jai-Hong Cheng[1,2,3], Wen-Yi Chou[1,4], Ching-Jen Wang[1,4], Ka-Kit Siu[1,4,5], Jei-Ming Peng[6], Yi-No Wu[7], Meng-Shiou Lee[8], Chien-Yiu Huang[1], Jih-Yang Ko[1,4*], Shun-Wun Jhan[1,4]*

[1]Center for Shockwave Medicine and Tissue Engineering, Kaohsiung Chang Gung Memorial Hospital and Chang Gung University College of Medicine, Kaohsiung, Taiwan.

[2]Department of Medical Research, Kaohsiung Chang Gung Memorial Hospital and Chang Gung University College of Medicine, Kaohsiung, Taiwan.

[3]Department of Leisure and Sports Management, Cheng Shiu University, Kaohsiung, Taiwan.

[4]Department of Orthopedic Surgery, Kaohsiung Chang Gung Memorial Hospital and Chang Gung University College of Medicine, Kaohsiung, Taiwan.

[5]Park One International Hospital, Kaohsiung, Taiwan.

[6]Institute for Translational Research in Biomedicine, Kaohsiung Chang Gung Memorial Hospital and Chang Gung University College of Medicine, Kaohsiung, Taiwan.

[7]School of Medicine, Fu Jen Catholic University, New Taipei City, Taiwan

[8]Department of Chinese Pharmaceutical Science and Chinese Medicine Resources, China Medical University, 91, Hsueh-Shih Road, Taichung, Taiwan.

*Correspondence:

Shun-Wun Jhan, M.D. Department of Orthopedic Surgery, Center for Shockwave Medicine and Tissue Engineering, Kaohsiung Chang Gung Memorial Hospital, 123 Dapi Road, Niao Sung District, Kaohsiung City 833, Taiwan. Email: b9502077@cgmh.org.tw

Jih-Yang Ko, M.D. Department of Orthopedic Surgery, Center for Shockwave Medicine and Tissue Engineering, Kaohsiung Chang Gung Memorial Hospital, 123 Dapi Road, Niao Sung District, Kaohsiung City 833, Taiwan. Email: kojy@cgmh.org.tw

Supplemental Table 1. The information of the experimental design.

Group Number	Group division	Group Name	OA induction	Positions
1	Sham	Sham	no	Without ESWT
2	OA	OA	yes	Without ESWT
3	Medial groups	M-T	yes	Medial tibia
4		M-F	yes	Medial femur
5		M-FT	yes	Medial femur and tibia
6		ML-T	yes	Medial lateral tibia
7	Lateral groups	L-T	yes	Lateral tibia
8		L-F	yes	Lateral femur
9		L-FT	yes	Lateral femur and tibia
10		LM-F	yes	Lateral medial tibia

Supplemental Table 2. The averages of the modified Mankin scores of femur and tibia in the groups**.**

Position	Groups				
	Sham	OA	M-T	M-F	M-FT
Femur	0.60±0.843*	27.60±2.12	11.00±3.43	17.13±4.12	7.88±2.90
Tibia	0.90±1.20	25.70±2.06	12.00±2.35	18.00±2.39	7.25±2.76

Position	Groups				
	ML-T	L-T	L-F	L-FT	LM-F
Femur	11.78±4.12	27.88±2.23	27.24±1.92	26.27±1.19	16.63±3.34
Tibia	10.89±4.37	27.63±1.20	27.00±1.85	27.00±2.26	16.88±2.23

*The modified Mankin score with a 0-33 scale.

Supplemental Table 3. The averages of tidemark roughness scores of femur and tibia in groups.

	Groups				
Position	Sham	OA	M-T	M-F	M-FT
Femur	1.29±0.05*	1.07±0.08	1.22±0.08	1.14±0.06	1.26±0.07
Tibia	1.311±0.06	1.06±0.06	1.17±0.09	1.08±0.03	1.24±0.07

	Groups				
Position	ML-T	L-T	L-F	L-FT	LM-F
Femur	1.19±0.05	1.05±0.07	1.06±0.07	1.06±0.08	1.15±0.07
Tibia	1.11±0.05	1.05±0.06	1.06±0.08	1.06±0.09	1.11±0.08

*The score of tidemark roughness with a minimum coefficient was at1.

Supplemental Table 4. The averages of calcified cartilage thicknesses of femur and tibia in groups.

	Groups[b]				
Position	Sham	OA	M-T	M-F	M-FT
Femur	59.37±16.45[a]	72.60±69.89	151.76±36.20	128.99±109.47	76.00±10.04
Tibia	83.65±12.61	74.20±70.69	188.13±24.02	161.38±110.28	106.02±14.26

	Groups				
Position	ML-T	L-T	L-F	L-FT	LM-F
Femur	136.37±45.68	54.91±49.77	76.49±79.46	30.16±40.43	58.77±67.03
Tibia	154.70±36.41	61.00±63.13	56.66±64.80	62.65±76.78	90.01±101.45

a. The score of calcified cartilage thicknesses with a minimum coefficient was at 0.

b. The high variations in the OA, M-F, L-T, L-F, L-FT and LM-F groups.
